# *Hypericum triquetrifolium*—Derived Factors Downregulate the Production Levels of LPS-Induced Nitric Oxide and Tumor Necrosis Factor-**α** in THP-1 Cells

**DOI:** 10.1093/ecam/nen056

**Published:** 2011-02-20

**Authors:** Bashar Saad, Bernadette Soudah AbouAtta, Walid Basha, Alaa Hmade, Abdalsalam Kmail, Said Khasib, Omar Said

**Affiliations:** ^1^Research and Development Regional Center—The Galilee Society, P.O. Box 437, Shefa Amr 20200, Israel; ^2^Qasemi Research Center—Al-Qasemi Academic College, Baga Algharbiya, Israel; ^3^Faculty of Allied Medical Sciences, Arab American University Jenin, P.O. Box 240, Jenin, Palestine; ^4^Antaki Center for Herbal Medicine Ltd, Kufur Kanna, Israel

## Abstract

Based on knowledge from traditional Arab herbal medicine, this *in vitro* study aims to examine the anti-inflammatory mechanism of *Hypericum triquetrifolium* by measuring the expression and release of pro-inflammatory cytokines, tumor necrosis factor-**α** (TNF-**α**) and interleukine-6 (IL-6), and inducible nitric oxide synthase (iNOS) in human monocytic cells, THP-1. The effects were assessed by measuring the levels of secretory proteins and mRNA of TNF-**α** and IL-6, the levels of nitric oxide (NO) secretion and the expression of iNOS in THP-1 cells. Cells were treated with 5 **μ**g lipopolysaccharide/ml (LPS) in the presence and absence of increasing concentrations of extracts from the aerial parts of *H. triquetrifolium*. During the entire experimental period, we used extract concentrations (up to 250 **μ**g mL^−1^) that had no cytotoxic effects, as measured with MTT and LDH assays. *Hypericum triquetrifolium* extracts remarkably suppressed the LPS-induced NO release, significantly attenuated the LPS-induced transcription of iNOS and inhibited in a dose-dependent manner the expression and release of TNF-**α**. No significant effects were observed on the release of IL-6. Taken together, these results suggest that *H. triquetrifolium* probably exerts anti-inflammatory effects through the suppression of TNF-**α** and iNOS expressions.

## 1. Introduction

In response to tissue injury, a multifactorial network of chemical signals initiate and maintain a host response designed to “heal" the afflicted tissue. This involves activation and directed migration of leukocytes (neutrophils, monocytes and eosinophils) from the venous system to sites of damage. Inflammation is the first response of the immune system to infection or irritation. It is caused by cytokines such as tumor necrosis factor-*α* (TNF-*α*, interleukin-1 (IL-1) and interleukin-6 (IL-6) [1, 2] and by eicosanoid such as PGE_2_ [3].

### 1.1. The Role of Proinflammatory Cytokines

Cytokines are regulators of host responses to infection, immune responses, inflammation and trauma. There are two types of cytokines: pro-inflammatory and anti-inflammatory. Thus, inhibitors of the pro-inflammatory cytokines have been considered as a candidate of anti-inflammatory drugs. Lipopolysaccharide (LPS)-activated macrophages are usually used for evaluating the anti-inflammatory effects of various materials. LPS is the principle component of the outer membrane of Gram-negative bacteria, is an endotoxin that induces septic shock syndrome and stimulates the production of inflammatory mediators such as nitric oxide (NO), TNF-*α*, interleukins, prostanoids and leukotrienes [4–6]. Therefore, LPS plays a key role in not only eliciting an inflammatory response but also in causing a septic shock during gram-negative bacterial infection. Inflammatory responses are advantageous for eradicating bacteria, as long as they are under control. When out of control, however, deregulated inflammation leads to the massive production of pro-inflammatory cytokines such as TNF-*α*, IL-1 and IL-6 by macrophages [1, 7], which can cause tissue injury and multiple organ failure [8]. For example, the resident macrophages of the liver, the Kupffer cells, are among the first to respond to foreign antigens. Activated hepatic Kupffer cells play an essential role in LPS-induced liver injury [9]. Following contact with the CD14 protein, the complex triggers a signal cascade involving nuclear factor kappa B (NF-*κ*B). This factor enhances the expression of inflammation-related genes. The acute-phase response is regulated by cytokines released by activated Kupffer cells, notably IL-1, IL-6, and TNF-*α* [10–12]. Among these cytokines, IL-6, also known as hepatocyte-stimulating factor, is a major inducer of the acute-phase response. In the liver, TNF-*α* production is not restricted to Kupffer cells. Saad et al. [13] have demonstrated that LPS affects the acute-phase response via hepatocyte-derived IL-6 and TNF-*α* in an autocrine loop and the NO production of parenchymal liver cells. TNF-*α* is also involved in inducing cell damage by promoting oxidative stress in mitochondria [14]. TNF-*α* stimulates the production of reactive oxygen species (ROS) and reactive nitrogen species. ROS have been implicated in the pathogenesis of many forms of liver disease. When liver cells are exposed to excesses of ROS, oxidative stress occurs and affects many cellular functions. The inflammatory process is controlled by immunosuppression cytokines such as interleukin-10 (IL-10) and interleukin-4. Macrophage-derived IL-10 affects the growth and differentiation of various cell types of the immune system *in vitro*. It inhibits the production of inflammatory cytokines such as IL-1, IL-6 and TNF-*α* by LPS-activated macrophages.

### 1.2. Medicinal Plants

Despite the great progress in modern medicine, traditional medicine has always been practiced in the Arab-Islamic world. Cultural beliefs and practices often led to self-medication, use of home remedies and consultation with traditional healers in rural areas. Traditional therapies have been utilized by people in the Mediterranean region, who have faith in spiritual healers, homeopaths or even many herbalists. These therapies are the first choice for people with problems such as infertility, impotence, diabetes, obesity, epilepsy, psychosomatic troubles and many other diseases. Arabic herbal medicine has played a remarkable role in curing inflammatory diseases in general and in clearly distinguishing between several subtypes of inflammatory diseases and has identified the curative properties of tens of plants for treating various types of inflammations [15–19].

### 1.3. *Hypericum triquetrifolium*

Herbal medicines containing *Hypericum triquetrifolium* have been used in traditional Arab herbal medicine to treat various inflammatory diseases. The classic Arabic name for this plant species is *Dathi* or *Nabtat Yohanna* [15–19]. Our previous studies show that *H. triquetrifolium* is not used any more within the practitioner communities in Galilee and in West Bank. This fact reflects an extinction process of important elements of the Arab herbal medicine heritage [15–19]. Utilizing the knowledge derived from traditional Arab herbal medicine and a recent *in vivo* report in which *H. triquetrifolium* extract exhibited anti-inflammatory activity in rats [20], this study explored the anti-inflammatory mechanism of *H. triquetrifolium*. Therefore, the expression and release of pro-inflammatory cytokines TNF-*α* and IL-6 and the inducible nitric oxide synthase (iNOS) in human monocytic cells, THP-1, were measured. Our results indicate that *H. triquetrifolium* could modulate the regulatory mechanism of NO and pro-inflammatory cytokines (TNF-*α* and IL-6) in the LPS-activated THP-1 cells. *Hypericum triquetrifolium* inhibited the production of NO and TNF-*α* and the expression of iNOS and TNF-*α* but not of IL-6.

## 2. Methods

### 2.1. Preparation of Plant Extracts

One-hundred grams of air-dried plant material was added to 1 l of distilled water and boiled for 10 min. The extract thus obtained was filtered using filter paper and frozen at −70°C until use. The frozen filtrate was freeze dried in a lyophilizer (48–72 hours). The freeze-dried extracts were stored at −70°C for further evaluation. These crude extracts were used for the following experiments.

### 2.2. Cell Culture

The human monocytic cell line THP-1 (ATCC 202-TIB) [21] was obtained from American Type Culture Collection (Manassas, VA, USA). These cells express various receptors that are found in normal monocytes and have been used as a model system for macrophage biology and leukemia since 1980. Cells were grown in Dulbecco's modified Eagle's medium with a high glucose content (4.5 g L^−1^), supplemented with 10% vol/vol inactivated fetal calf serum, 1% non-essential amino acids, 1% glutamine, 100 U mL^−1^ penicillin and 10 *μ*g mL^−1^ streptomycin. Cell lines were maintained in a humidified atmosphere of 95% O_2_–5% CO_2_ at 37°C. The culture medium was changed twice a week. Cells were seeded in 24-well plates at a density of 2 × 10^5^ cells mL^−1^. Cells were activated with PMA (100 ng mL^−1^) and Vitamin D3 (0.1 *μ*M). Twenty-four hours after cell activation, cells were exposed to varying concentrations of the plant extracts in a fresh serum-free medium in both the presence and absence of LPS (5 *μ*g mL^−1^).

### 2.3. MTT Assay

The tetrazolium dye, MTT, is widely used to assess the viability and/or the metabolic state of the cells [22]. The MTT-colorimetric monocyte mediated cytotoxicity assay, based upon the ability of living cells to reduce 3-[4,5-dimethylthiazol-2-yl]-2,5 diphenyltetrazolium bromide (MTT) into formazan by mitochondrial succinate dehydrogenase in viable cells. Twenty-four hours after cell seeding, cells were incubated with varying concentrations of water extracts of *H. triquetrifolium* for 24 hours at 37°C. Following the removal of the plant extracts from each well, cells were washed in phosphate-buffered saline (PBS). The cells were then incubated in serum-free RPMI to which MTT (0.5 mg mL^−1^) was added to each well (100 *μ*L) and incubated for a further 4 hours. Then the medium was removed and the cells were incubated for 15 min with 100 *μ*L of acidic isopropanol (0.08 N HCl) to dissolve the formazan crystals. The absorbance of the MTT formazan was determined at 570 nm in an enzyme-linked immunosorbent assay (ELISA) reader. Viability was defined as the ratio (expressed as a percentage) of absorbance of treated cells to untreated cells.

### 2.4. Lactate Dehydrogenase

In the lactate dehydrogenase (LDH) assay, the leakage of the cytoplasm-located enzyme LDH into the extracellular medium is measured. The presence of the exclusively cytosolic enzyme, LDH, in the cell culture medium was indicative of cell membrane damage [23].

For the LDH assay, 5 × 10^3^ THP-1 were seeded per well in 96-well microtiter plates. Twenty-four hours after cell seeding, cells were exposed to varying concentrations of *H. triquetrifolium* extracts (0–500 *μ*g mL^−1^). After 24 hours of treatment, the supernatants were collected from each well. Cell monolayers were then treated with a cell lysis solution for 30 minutes at room temperature. The cells and the lysate were collected. LDH activity was measured in both the supernatants and the cell lysate fractions using CytoTox 96, a non-radioactive cytotoxicity assay kit (Promega, WI, USA), in accordance with the manufacturer's instruction. The absorbance was determined at 490 nm using 96-well plate ELISA reader. The percent of LDH released from the cells was determined using the formula: LDH release = (absorbance of the supernatant)/(absorbance of the supernatant and cell lysate) × 100.

### 2.5. Nitrite Determination

Nitrite determinations were carried out in 50 *μ*l aliquots of sample mixed with 200 *μ*l of the Griess reagent [24].

### 2.6. Quantification of TNF-*α* Production

TNF-*α* levels were determined in TNF-*α*-specific bioassay using WEHI cell line, as previously described [25]. WEHI 164 clone 13 fibrosarcoma cells at a concentration of 2 × 104 cells per 100 *μ*L were incubated with serially diluted samples in 96-well flat bottom microtiter plate for 48 hours at 37°C, 5% CO_2_. Then 10 *μ*L of a 5 mg mL^−1^ MTT tetrazolium solution in PBS was added to the plate, which was further incubated for 4 hours. The dye was then removed and cells were lysed by addition of 100 *μ*L of isopropanol-5% formic acid. Plates were read at 620 nm on a multiscan bichromatic ELISA reader (Flow Labs).

### 2.7. Immunoassay for Cytokines

Commercial ELISA kits (R&D Systems, Minneapolis, MN, USA) were used to quantify TNF-*α* and IL-6. The absorbance at 450 nm was read by a microplate reader (model 680; Bio-Rad Laboratories, Mississauga, ON, Canada), with the wavelength correction set at 550 nm. To calculate the concentration of TNF-*α* and IL-6, a standard curve was constructed using serial dilutions of cytokine standards provided with the kit.

### 2.8. Reverse Transcriptase-Polymerase Chain Reaction Analysis

Samples were centrifuged at 1000 g for 10 min and total RNA was prepared using Master pure RNA purification kit (EPICENTRE Biotechnologies). Reverse transcriptase-polymerase chain reaction (RT-PCR) was performed using the Master pure RNA purification kit system (ABgene). Total RNA (0.1 *μ*g) was used for a single reaction. Nucleotide sequences of oligonucleotide primers for the housekeeping glyceraldehyde-3-phosphate dehydrogenase (G3PDH) plus the TNF-*α* or iNOS primer pairs were used for RT-PCR, which are described elsewhere [26]. The reverse transcriptase reaction was performed at 55°C for 30 minutes. To amplify the G3PDH TNF-*α* and iNOS cDNA, each sample was denatured at 95°C for 60 s, annealed at 55°C for 60 s and extended at 72°C for 90 s. The RT-PCR products were subjected to agarose gel electrophoresis and stained by ethidium bromide.

### 2.9. Statistical Analysis

Error limits cited and error bars plotted represent simple standard deviations of the mean. When comparing different samples, results were considered to be statistically different when *P* < .05 (Student's *t*-test for unpaired samples).

## 3. Results

### 3.1. Toxicity Measurements

The anti-inflammatory effects of *H. triquetrifolium* were evaluated here in cells from the human monocyte cell line. MTT and LDH assays were carried out in order to evaluate non-toxic concentrations of *H. triquetrifolium.*

### 3.2. MTT Test

**Figure 1:**
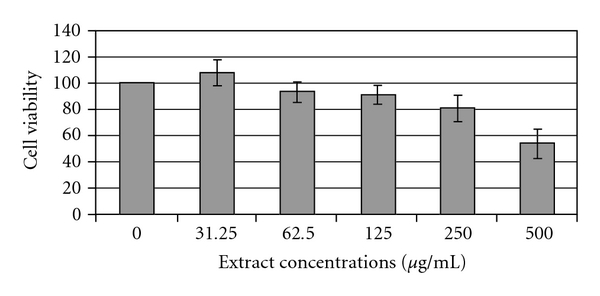
MTT assay in THP-1 cells after overnight treatment with varying concentrations of extract from *H. triquetrifolium*. The absorbance of the MTT formazan was determined at 570 nm using ELISA reader. Cell viability was defined as the ratio (expressed as a percentage) of absorbance of treated cells to untreated cells. Values represent mean ± SD (*P < .05 significant as compared with controls) of three independent experiments carried out in triplicate.

The metabolic activity can be evaluated by measuring the activity of a mitochondrial enzyme succinate dehydrogenase using the MTT test. This test is widely used in the *in vitro* evaluation of the toxicity of plant extracts. We applied the MTT test to evaluate the safety of extracts from *H. triquetrifolium* in cells from the human monocyte cell line THP-1. Cells were exposed to increasing concentrations (1–500 *μ*g mL^−1^ of culture medium) of *H. triquetrifolium* extracts for 24 hours. No sign of any negative effects was observed after treatment with concentrations up to 250 *μ*g mL^−1^ (Figure 1). Concentrations higher than 250 *μ*g mL^−1^ caused a significant reduction in the cell viability.

### 3.3. LDH Release Test

**Figure 2:**
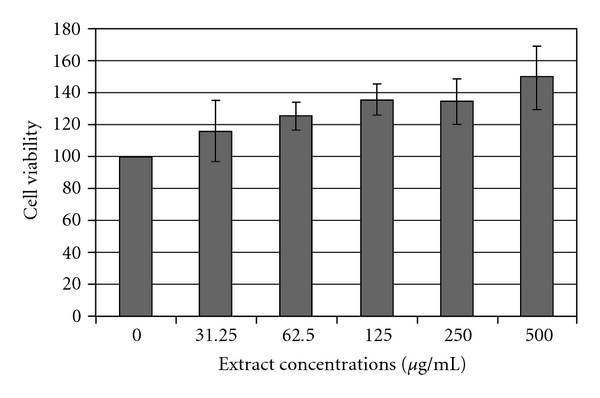
LDH leakage from THP-1 cells after overnight incubation with varying concentrations of extracts from *H. triquetrifolium*. The leakage of the cytoplasm-located LDH into the extracellular medium is measured. LDH activity was measured in both the supernatants and the cell lysate fractions. Values given represent the mean ± SD (*P < .05 significant as compared with controls) of three independent experiments carried out in triplicate.

Membrane integrity can be evaluated by measuring LDH activity. LDH, an enzyme located in the cytoplasm, catalyses the conversion of lactate and pyruvate. When LDH is found in the media of the cells, there are two possible causes: the first is cellular death and the second is a “leak" in a cell membrane. When cells are disrupted, the LDH activity is elevated. Results obtained indicate no significant changes in LDH levels in the culture medium after exposure to extracts of *H. triquetrifolium* at concentration up to 125 mg mL^−1^. A slight, but not significant, increase was observed after treatment with concentration of 250 and 500 *μ*g mL^−1^ (Figure 2).

Based on the MTT and LDH results, we decided to exclude concentrations of 500 mg mL^−1^ and to use concentrations below 250 *μ*g mL^−1^ in the following experiments.

### 3.4. LPS-Induced No Production

**Figure 3:**
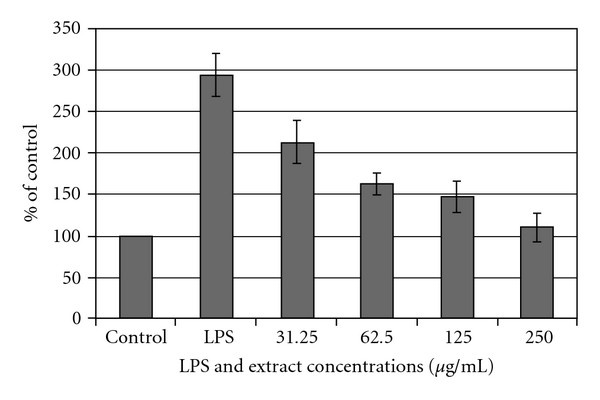
Dose-dependent inhibition of LPS-mediated production of NO by *H. triquetrifolium* extracts. For each concentration treatment, the level of NO release is represented as a percentage of the control set at 100%. Values represent mean ± SD (*P < .05 significant as compared with LPS alone) of three independent experiments carried out in triplicate.

Figure 3 shows the dose-dependent inhibition of the LPS-mediated production of NO by *H. triquetrifolium* extracts. *Hypericum triquetrifolium* extracts inhibited NO production by cultured THP-1 in a dose-dependent manner, reaching the control levels of untreated cells at a concentration of 250 *μ*g mL^−1^ (Figure 3).

### 3.5. Pro-Inflammatory Cytokines IL-6 and TNF-*α*

Production of IL-6 and TNF-*α* by cultured THP-1 was tested in the culture supernatants using commercial ELISA kits. It was found that THP-1 produced detectable amounts of IL-6 and TNF-*α* after stimulation with LPS. Maximal TNF-*α* and IL-6 concentrations were detectable in the culture supernatants 4 and 6 hours after LPS stimulation, respectively. Therefore, the 4 and 6 hours time points were used to characterize the effects of *H. triquetrifolium* extracts on TNF-*α* and IL-6 production by cultured THP-1, respectively.

**Figure 4:**
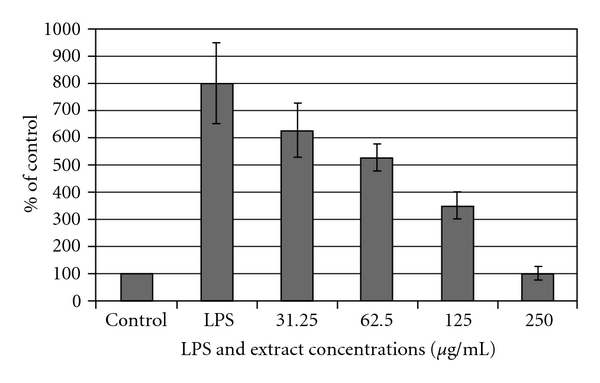
Dose-dependent inhibition of LPS-mediated production of TNF-*α* by *H. triquetrifolium* extracts. For each concentration treatment, the level of TNF-*α* release is represented as a percentage of the control set at 100%. The bar heights represent the values of mean ± SD (*P < .05 significant as compared with LPS alone) of three independent ELISA experiments carried out in triplicate.

Figure 4 shows the TNF-*α* secretion into the culture supernatant of untreated and LPS-treated THP-1. *Hypericum triquetrifolium* extracts inhibited TNF-*α* production in a dose-dependent manner, reaching the control levels of untreated cells at a concentration of 250 *μ*g mL^−1^ (Figure 4). No effects on the production levels of IL-6 were seen after LPS treatment in the presence of *H. triquetrifolium* (data not shown).

### 3.6. TNF-*α* Gene Expression

**Figure 5:**
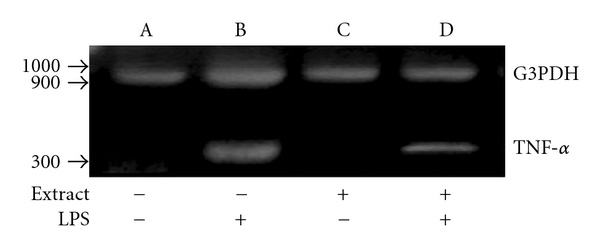
*Hypericum triquetrifolium* extract suppresses LPS-induced TNF-*α* gene expression in human THP-1 cells. RNAs from LPS-treated, extract-treated or LPS plus extract-treated THP-1 cells were reverse transcribed and amplified with human G3PDH and TNF-*α* primer pairs and electrophoresed. G3PDH and TNF-*α* messages amplified at around 983 and 325 bp, respectively. Extract demonstrated a significant suppression of endogenous TNF-*α* message (lane D) compared with that in the LPS-treated culture (lane B). These results are from a single experiment, which was repeated three times.

We further studied the effect of *H. triquetrifolium* on TNF-*α* mRNA in LPS-activated THP-1 cells in the presence and absence of varying concentrations of *H. triquetrifolium*. In this system, reverse-transcribed RNA products were amplified simultaneously with both internal control gene primers (G3PDH) and TNF-*α* primers in the same tube. Data presented in Figure 5 demonstrate that both amplified human G3PDH gene (983 bp) and TNF-*α* gene (325 bp) products amplified at the expected regions. Cells cultured with *H. triquetrifolium* did not produce any significant effect on TNF-*α* mRNA. However, the extract significantly suppressed the LPS-induced TNF-*α* message compared with expression in the LPS-treated culture.

### 3.7. INOS Gene Expression

**Figure 6:**
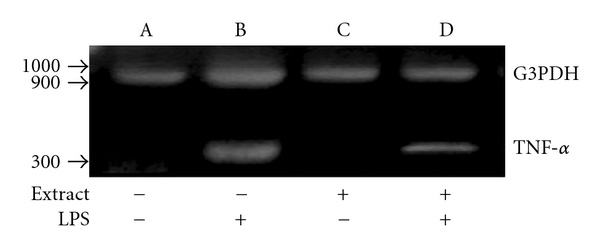
*Hypericum triquetrifolium* extract suppresses LPS-induced iNOS gene expression in human THP-1 cells. RNAs from LPS-treated, extract-treated or LPS plus extract-treated THP-1 cells were reverse transcribed and amplified with human G3PDH and iNOS primer pairs and electrophoresed. Extract demonstrated complete suppression of endogenous iNOS message (lane D) compared with that in the LPS-treated culture (lane B). These results are from a single experiment, which was repeated three times, using THP-1 cells.

Figure 6 shows that treatment of THP-1 cells with extracts of *H. triquetrifolium* completely suppressed the LPS-induced iNOS gene expression. Data presented in Figure 6 demonstrate that both amplified human G3PDH gene (983 bp) and iNOS gene (300 bp) products amplified at the expected regions. Cells cultured with *H. triquetrifolium* did not produce any significant effect on iNOS mRNA. The extract, however, totally suppressed the LPS-induced iNOS message compared with expression in the LPS-treated culture (Figure 6).

## 4. Discussion

Inflammation is the first response of the immune system to infection or irritation. It is caused by cytokines such as TNF-*α*, IL-1 and IL-6 and by eicosanoid such as PGE_2_. Thus, inhibitors of these cytokines have been considered as a candidate for anti-inflammatory drugs. Monocytes/macrophages are key mediators of inflammation and are widely distributed in the body [27, 28]. Therefore, the monocytic cell line THP-1, which represents an appropriate model system to study immune responses, was utilized to investigate the anti-inflammatory effects of *H. triquetrifolium*.

Herbal medicines containing *H. triquetrifolium* have been used in traditional Arab herbal medicine to treat various inflammatory diseases. However, only few studies have been conducted to evaluate the effects of *H. triquetrifolium* on inflammation. In this study, we show that *H. triquetrifolium* could modulate the regulatory mechanism of NO and pro-inflammatory cytokines (TNF-*α* and IL-6) in the LPS-activated THP-1 cells. *Hypericum triquetrifolium* inhibited the production of NO and TNF-*α* and the expression of iNOS and TNF-*α* but not of IL-6.

### 4.1. The Effect on NOS

NO is a free radical produced from l-arginine by nitric oxide synthases (NOSs). It is also an important cellular second messenger. NO is one of the cellular mediators of physiological and pathological processes [21, 29]. At adequate concentrations, NO can generate or modify intracellular signals, thereby affecting the function of immune cells, tumor cells and resident cells of different tissues and organs. However, its uncontrolled release can cause inflammatory destruction of target tissue during an infection. Three different isoforms of NOS have been discovered: endothelial NOS, neuronal NOS and iNOS. The former two are constitutively expressed in the body, whereas the latter is an inducible enzyme highly expressed by inflammatory stimuli in certain cells such as macrophages. A significantly increased amount of NO synthesized by iNOS participates in provoking inflammatory process and acts synergistically with other inflammatory mediators. Inhibition of iNOS activity or downregulation of iNOS expression may be beneficial to reduce the inflammatory response. Therefore, it is meaningful to evaluate the effects of extracts from *H. triquetrifolium* on NOS (effect on NO production) since NO is one of the inflammatory mediators. Our results demonstrate for the first time that *H. triquetrifolium* extracts downregulate the production of NO by downregulating the transcription of the iNOS gene. The transcription was totally inhibited by non-toxic concentration of *H. triquetrifolium* extract (250 *μ*g *H. triquetrifolium*/mL). Similar results were found with medicinal plant-derived factors (e.g., flavonoids). Using LPS/cytokine-treated macrophages or macrophage-like cell lines, varieties of flavonoids including apigenin, luteolin and quercetin were found to inhibit NO production. However, studies on the mechanism have shown that flavonoids did not significantly inhibit iNOS. They were shown to downregulate iNOS induction, thereby reducing NO production [30]. Recently, Moutan Cortex, a traditional medicine used to remove heat from the blood, promote blood circulation and alleviate blood stasis, has been found to exhibit anti-inflammatory effects through the inhibition of iNOS and COX-2 expression by suppressing the phosphorylation of I-*κ*B*α* and the activation of NF-*κ*B [31].

### 4.2. The Effect on the Production of Pro-Inflammatory Cytokines

In addition to iNOS, several cytokines are intimately associated with inflammatory diseases. In particular, TNF-*α* and IL-1 are prominent contributors to chronic inflammatory disorders including rheumatoid arthritis (RA) [32]. TNF-*α* and IL-1 receptor antagonists have been clinically successful in improving the symptoms in RA patients. SAIDs, such as prednisolone and dexamethasone, are known to reduce the production of these cytokines. In recent years, various medicinal plant-derived factors have been reported to regulate the production of pro-inflammatory cytokines. Flavonids, such as amoradicin, genistein and silybin, were proved to inhibit TNF-*α* production from LPS-treated RAW 264.7 cells [33]. Baicalin inhibited the induction of IL-1, IL-6, TNF-*α*, interferon-*γ*, monocyte chemotactic protein-1, macrophage inflammatory protein (MIP)-1 and MIP-1 at protein as well as at RNA levels from human blood monocytes treated with staphylococcal enterotoxin [34]. In this study, we have shown that *H. triquetrifolium* inhibits the production of LPS-induced TNF-*α* production by downregulating the transcription of the TNF gene. Similar results were found using different medicinal plants. For example, feverfew extracts were found to effectively reduce LPS-mediated TNF-*α* and CCL2 (MCP-1) releases by THP-1 cells [28]. Moutan Cortex extracts were reported to exhibit anti-inflammatory effects through the inhibition of iNOS and COX-2 expression by suppressing the phosphorylation of I-*κ*B*α* and the activation of NF-*κ*B. *Uncaria tomentosa* extracts inhibited the MAP kinase-signaling pathway and altered cytokine expression in THP-1 monocyte-like cells with *U. tomentosa* [31]. *Hypericum triquetrifolium* inhibits the production of LPS-induced TNF-*α* production by downregulating the transcription of the TNF gene but has no significant effects on the production levels of IL-6. These results suggest that the anti-inflammatory effects of *H. triquetrifolium* are mediated via the downregulation of the transcription of the TNF gene.

## 5. Conclusions

Results obtained in this *in vitro* study indicate that extracts from *H. triquetrifolium* inhibit the production of LPS-induced TNF-*α* and NO production by downregulating the transcription of TNF-*α* and NO genes, respectively.
